# Poly(sulfur nitride) Nanowires as Short Circuits between Highly Oriented Pyrolytic Graphite Sheets

**DOI:** 10.1021/acs.langmuir.6c03183

**Published:** 2026-09-02

**Authors:** Jörg Kressler, Karsten Busse, Julian Schoch-Radicke, Oleksandr Dolynchuk, Stefan G. Ebbinghaus, Frank Syrowatka

**Affiliations:** † Faculty of Natural Science II, 9176 Martin Luther University Halle-Wittenberg, Halle (Saale) D-06120, Germany; ‡ Interdisciplinary Center of Materials Science, Martin Luther University Halle-Wittenberg, Halle (Saale) 06099, Germany

## Abstract

Highly oriented pyrolytic graphite (HOPG) is intercalated with poly­(sulfur nitride) (SN)_*x*_. In a first step, HOPG is swollen in *N*-methyl pyrrolidone (NMP) supported by ultrasonication. Then, HOPG is exposed to disulfur dinitride (S_2_N_2_) vapor, which polymerizes to (SN)_*x*_ nanowires oriented perpendicular to the HOPG platelets, with a thickness of roughly 30 nm. The graphene layers constituting the platelets remain largely intact. The S_2_N_2_ ring-opening polymerization is mainly initiated at the lateral edges of the HOPG mosaic crystals, as observed by scanning electron microscopy. The electrical short circuit between the HOPG platelets, caused by the electrically conductive (SN)_*x*_ nanowires, increases the diamagnetism parallel to the basal HOPG planes, which is perpendicular to the (SN)_*x*_ nanowires. This results in a strong decrease in the diamagnetic anisotropy, defined as the ratio of the real part of the susceptibility parallel $\chi_{∥}^{\prime }$ and perpendicular $\chi_{⊥}^{\prime }$ to the crystallographic *c*-axis of HOPG. Thus, the ratio $\chi_{∥}^{\prime }$ / $\chi_{⊥}^{\prime }$ ≥ 40 for pure HOPG drops to $\chi_{∥}^{\prime }$ / $\chi_{⊥}^{\prime }$ ∼ 3 after intercalation with (SN)_*x*_.

## Graphical abstract

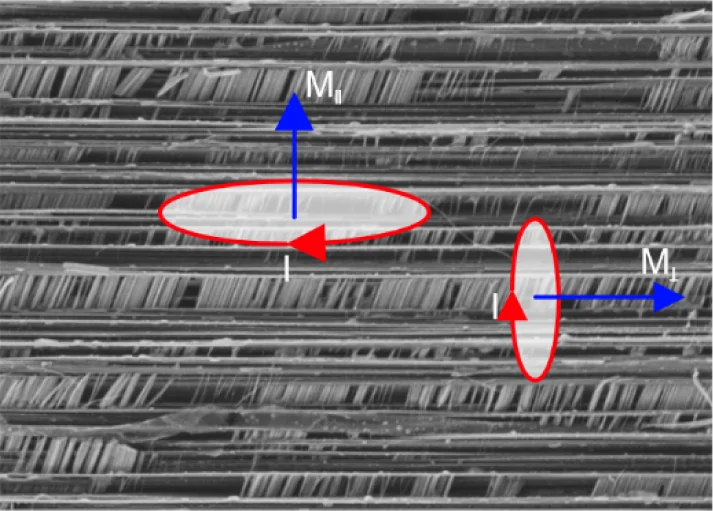

## Introduction

In the predominant literature, bulk samples of highly oriented pyrolytic graphite (HOPG) are defined as an anisotropic semimetal if considered parallel or perpendicular to the carbon planes.^1,2^ Some major applications include, e.g., monochromators for X-rays and neutrons;^3^ substrates for graphene production by mechanical, solvent, or electrochemical exfoliation;^4^ electrode materials;^5,6^ fuel cells;^7^ supercapacitors;^8^ and magnetic levitation (MagLev).^9^ HOPG is a well-organized material over several length scales. A network of hexagonally ordered sp^2^-hybridized carbon atoms is arranged in the crystallographic a-plane (note that for HOPG, a = b), also called the basal plane, having a distance of 1.42 Å (see Figure 1a). The graphene layers with a distance of 3.354 Å from each other are stacked in an ABA (Bernal-type) mode with *P*6_3_/*mmc* symmetry^10,11^ or as a minor component in an ABCA (rhombohedral-lattice) mode with *R*3*m* symmetry.^12^ They are arranged in shallow mosaic crystals with a typical thickness of about 30 nm and a lateral size of 6 to 30 μm (Figure 1b).^13^ These mosaic crystals are organized into sheets caused by the HOPG production process (Figure 1c).^14^ The nonperfect parallel arrangement of these sheets is characterized by the mosaicity, with a mosaic spread usually in the range between 0.2 and 0.8°^15^ or smaller than 0.1° for highly annealed pyrolytic graphite (HAPG).^16^ On the (00l) top layers, small terraces can be observed by AFM/STM^17^ or by SEM, as discussed below.

**Figure 1:**
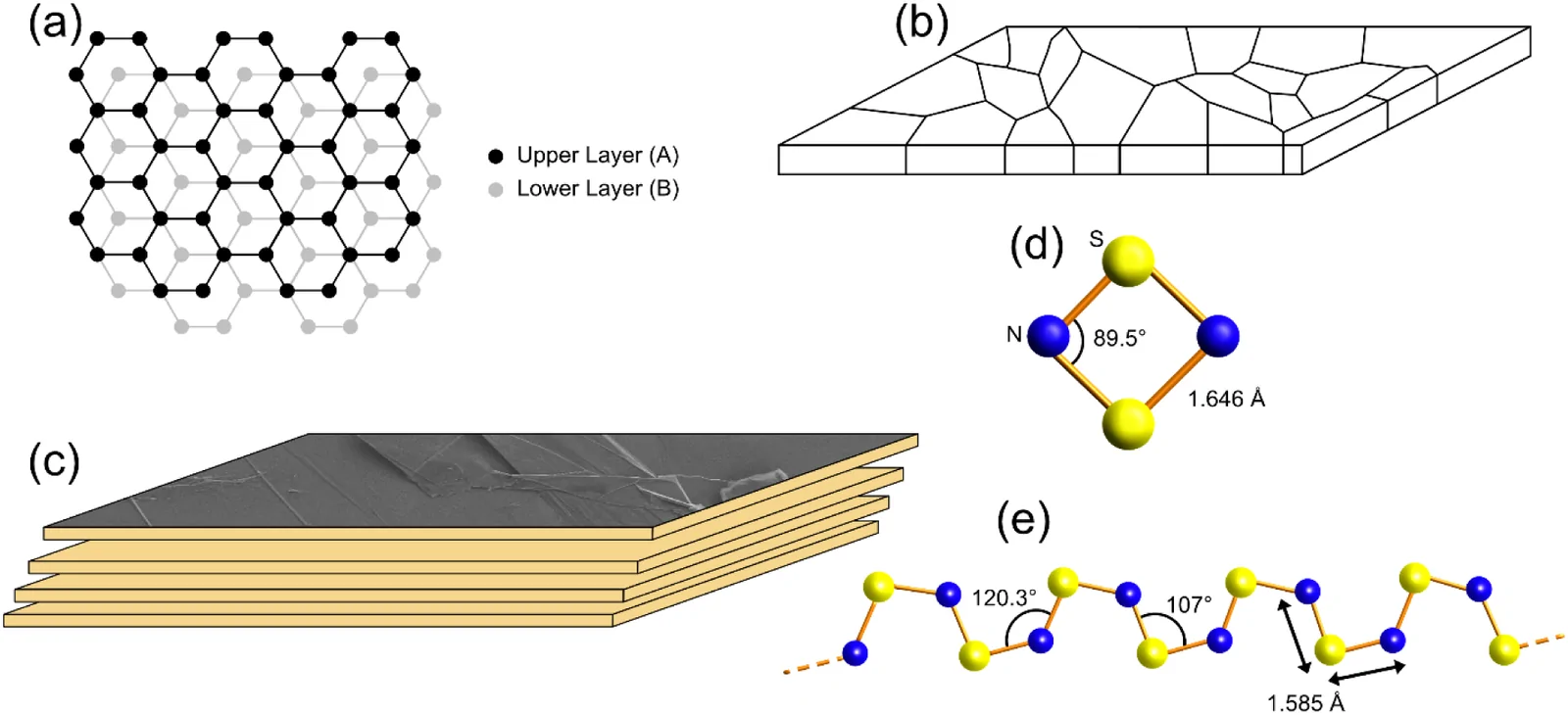
(a) Two AB graphene layers with a honeycomb structure of carbon atoms. The top and bottom edges have armchair conformations and the left and right edges show zigzag conformations. (b) Polycrystalline HOPG sheets with shallow mosaic crystals formed by several parallel graphene layers. (c) Parallel arrangement of graphite sheets. (d) Molecular structure of S_2_N_2_. (e) Polymer chain segment of (SN)*_x_*.

Within the graphene layers, the carbon atoms are linked not only by directed sp^2^–sp^2^ bonds but also by unhybridized, perpendicular p-electrons. This is the basis for highly delocalized π-electrons in the basal a-plane, resulting in a large electrical conductivity at room temperature, depending on the crystal perfection.^18^ This conductivity is several orders of magnitude larger compared to the one in the crystallographic c-direction. This results simultaneously in a strong anisotropy of the magnetic properties. HOPG is as any known carbon modification considered to be diamagnetic, i.e., it generates a magnetism opposing an applied external magnetic field.^19^ HOPG has the strongest diamagnetism of any known material at room temperature (RT) and ambient pressure, with a susceptibility in the c-direction of *χ*_||_ = −21.5 × 10^–6^ emu/g at RT^20^ and −30 × 10^–6^ emu/g below 100 K^19^ obtained by direct current (DC) susceptibility measurements. In the perpendicular direction, the susceptibility χ_⊥_ is −0.5 × 10^–6^ emu/g, about 40 to 60 times smaller. Yet, this value is several orders of magnitude smaller than χ for superconductors below their critical temperature *T*_c_, which is in the ideal case −1 emu/g (diamagnetic Meißner effect).^21^ The diamagnetic in-plane susceptibility of HOPG is close to zero or might even be positive^22^ The experimentally obtained small negative value might be the result of the nonperfect ordering of HOPG.

Since the lamellar-ordered graphene planes of HOPG are mainly kept together by van der Waals forces, it is possible to swell, intercalate, or exfoliate them.^23,24^ Here, we apply liquid swelling of HOPG in NMP, followed by disulfur dinitride (S_2_N_2_; Figure 1d) intercalation together with polymerization to poly­(sulfur nitride) (SN)_*x*_ (Figure 1e). The physical properties of (SN)_*x*_ show a strong anisotropy, too. (SN)_*x*_ is also a semimetal with a conductivity at room temperature of (1–4) × 10^3^ S cm^–1^ along the fiber direction depending on the quality of (SN)_*x*_ crystals.^25,26^ The conductivity increases by a factor of 10^2^ when the sample is cooled to 4.2 K.^27^ It is the only synthetic polymer that exhibits superconductivity (below 0.26 K).^28^ Both properties, i.e., conductivity and superconductivity, occur without any doping, which is unique in the field of synthetic conductive polymers. The charge-carrier mobilities along and perpendicular to the fiber direction are 250 and 6 cm^2^ V^–1^ s^–1^, respectively,^29^ which are much smaller than those of HOPG.^30^

In the present study, HOPG was swollen in NMP and subjected to a stream of S_2_N_2_ gas under ultrahigh vacuum conditions at ice-water temperature. At this temperature, S_2_N_2_ polymerizes slowly to (SN)_*x*_. This leads to (SN)_*x*_ intercalation, which alters the magnetic properties of HOPG considerably, especially in the basal plane. The anisotropy of the magnetic properties together with the morphology of the HOPG + (SN)_*x*_-system are considered. It should be noted that this procedure is fundamentally different to the so-called sublimation of (SN)_*x*_ onto polymeric substrates^31,32^ or graphite^33^ in vacuum at about 350 K. The frequently employed term “sublimation” is not correct and should be replaced by depolymerization-repolymerization of intermediate species different from S_2_N_2_.^34^ There is one report where S_2_N_2_ is polymerized into the zeolite channels of Na-ZSM-5 with a 5.5 Å diameter, without achieving any electrical conductivity.^35^ Our current study provides detailed information on the preparation of a new material morphology when HOPG is intercalated with (SN)_*x*_. This combination of two semimetals is closely interconnected with new magnetic properties with potential applications in nanotechnology.

## Results and Discussion

The cutting procedure for the commercial HOPG specimen (10 mm × 10 mm × 1 mm) and the cutting to square-shaped samples (5 mm × 5 mm × 1 mm) by ourselves results in edge regions ⟨hk0⟩ where the ordering of shallow graphite crystallites parallel to the basal plane is lost in the range of several micrometers (see SEM image in Figure 2a). This is also confirmed by spatially resolved microfocus 2D-WAXS measurements (Figure 2b–d). The Laue condition for the (002) reflection is not fulfilled for transmission measurements parallel to the surface normal if the cut edges are not involved. WAXS transmission measurements in the edge regions unambiguously show that in the X-ray spot region of the measurement, the HOPG crystallites are tilted in the cutting direction, and the (002) reflection occurs (Figure 2b) from the front side and in Figure 2c from the left side of the HOPG specimen shown in Figure 2a. Figure 2d taken from the center region of the HOPG sample shows very high ordering of the carbon layers as characterized by the double Bragg reflection in the small-angle region.^36^ The complete imaging by WAXS transmission measurements is given in the Supporting Information, Figure S1. It indicates that the center region of the HOPG specimen retains a highly ordered HOPG layer system even after swelling and S_2_N_2_ polymerization. Simultaneously, the (00l) surface remains relatively smooth and is covered with some (SN)_*x*_ crystals. A typical SEM image of such a crystal is shown in Figure 2e. These crystals are generally nucleated on small HOPG surface terraces and not on the basal planes of HOPG, as will be discussed below. The (SN)_*x*_ crystal formation occurs usually by topochemical solid-state polymerization in preformed S_2_N_2_ crystals.^37^ These crystals actually serve as a monomer reservoir for the polymerization process. This is the reason for the formation of fibrous (SN)_*x*_ crystals having sharp contours and smooth surfaces in images obtained by polarized optical microscopy (POM).^38^ These crystals are not strongly attached to the HOPG (00l) surface and can be removed mechanically without leaving any trace on the HOPG (00l) surface. These results are also confirmed by WAXS measurements in the reflection mode (Figure 2f). The HOPG lamellar system provides the (002) and the (004) reflections. The full width at half maximum (fwhm) values are similar to those of the original HOPG samples, which already show some deviations between different batches. The nonoccurrence of the (100) reflection at 2Θ = 42.5° indicates that the cutting of the HOPG specimen does not lead to large deformed areas of the specimen. This is different from a specimen machined from graphite platelets.^39^ The inset depicts a 2D WAXS image of an (SN)_*x*_ crystal attached to HOPG. It shows the (002) reflex of HOPG, together with the (102̅) reflex and the (100) reflex of the predominant β phase of (SN)_*x*_ within the circular structure. Outside the circular structure, the (110) and (012) reflexes can be identified.^40^ The (SN)_*x*_ structures formed on the edge surfaces are obviously grown by a different mechanism, as depicted in Figure 2g. In very rare cases, crystals with some similarities to (SN)_*x*_ bulk samples can be observed. Instead of fibrous crystals that are formed in the dimensions of the preformed S_2_N_2_ crystals, the (SN)_*x*_ crystal shows some HOPG fragments on its surface, as confirmed by Raman microscopy. It is obvious that the crystal growth must start in the interlamellar HOPG regions. The typical Raman signals of (SN)_*x*_ at 473, 670, and 799 cm^–1^ are assigned in agreement with literature data (Figure 2h).^41^ The Raman signals of HOPG on the (SN)_*x*_ surface occur at 1581 and 2721 cm^–1^. Raman spectra taken on the same sample but not on top of an (SN)_*x*_ crystal appear at 1583 and 2727 cm^–1^ (Supporting Information, Figure S2). The signal at about 1580 cm^–1^ is the G peak related to the E_2g_ phonon at the Brillouin zone center.^42^ The D peak, caused by the breathing mode of sp^2^ carbon atoms, is not observed since it would require defect activation.^43^ But its second-order 2D peak at about 2720 cm^–1^ does not require defect activation and can therefore be observed. Due to the multilayer system of HOPG, this is a broad peak in contrast to single-layer graphene. Basically, it is possible to calculate the number of graphene layers from the fwhm of the 2D signal or from the intensity ratio of I­(2D)/I­(G). The back gating as well as the top gating of the G peak at different sample positions are related to the state of doping of graphite and will not be discussed in more detail here. As already described in the literature,^44^ there might be a broad distribution of these values within a single sample specimen. Another morphology in different areas of the (hk0) surfaces of the nonswollen HOPG sample exposed to S_2_N_2_ vapor can be seen in Figure 2i and 2j. Single nanowire bundles are observed in the SEM image of Figure 2i. This type of morphology has also been observed when S_2_N_2_ is polymerized into pores of MgB_2_, and the elemental composition was determined by energy-dispersive X-ray spectroscopy (EDX).^45^ The growth of single (SN)_*x*_ nanowire bundles rather than crystals is caused by a higher nucleation density, as will be discussed below. Figure 2j depicts a large area with graphite layers in the top region, as expected from the ideal structure of HOPG given in Figure 1c. Since intercalation with (SN)_*x*_ in this area cannot be observed, it seems reasonable to swell HOPG prior to S_2_N_2_ polymerization.

**Figure 2:**
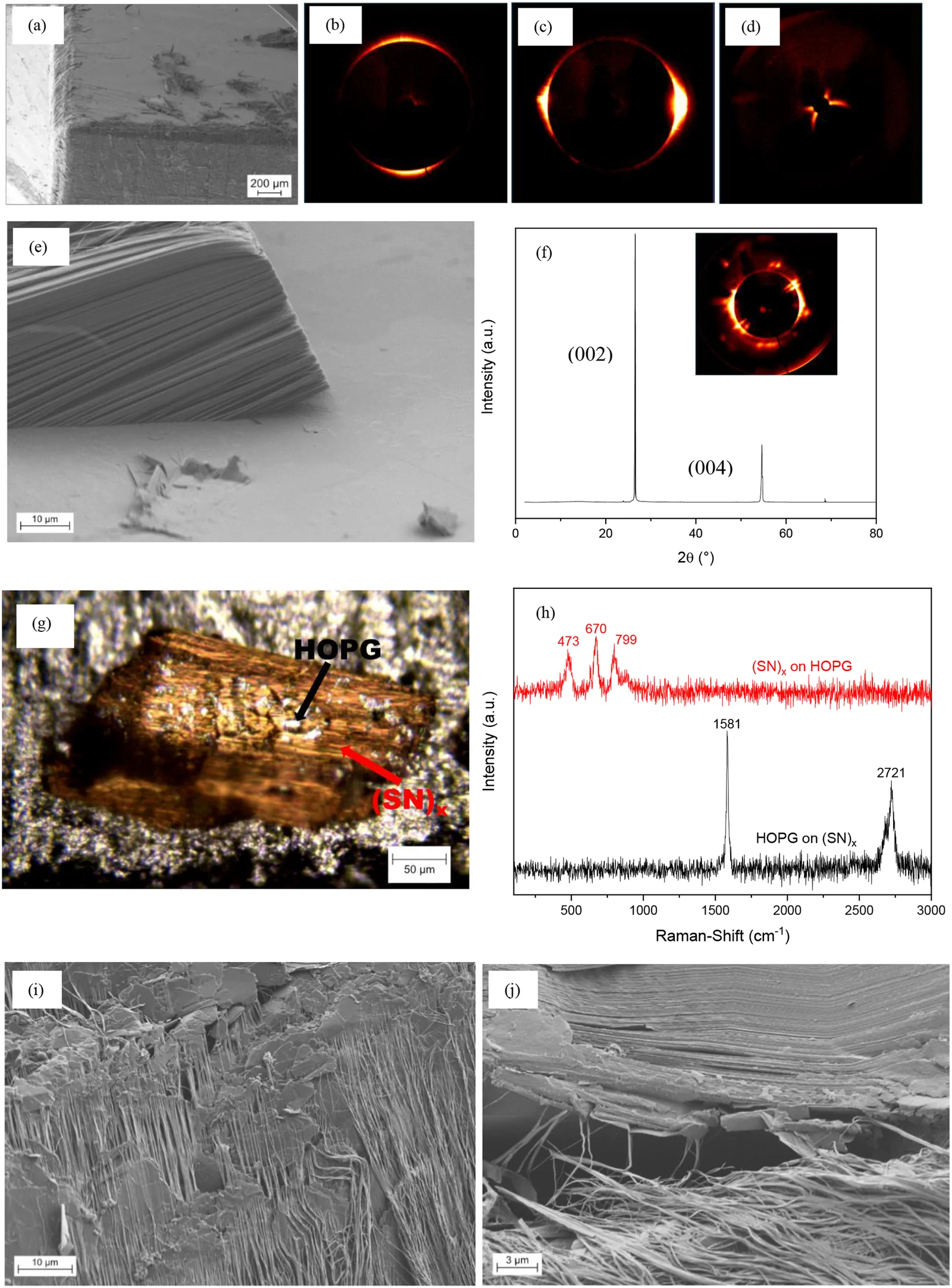
(a) SEM image of HOPG after exposure to S_2_N_2_ vapor. (b) 2D-WAXS image in the transmission mode at a spot of the front side of (2a). (c) 2D-WAXS image measured at a spot of the left side of (2a). (d) 2D-WAXS image taken at a spot from the center region of (2a). (e) SEM image of an (SN)*_x_* crystal grown on the HOPG (00l) surface. (f) WAXS measurement of an HOPG specimen taken in the reflection mode. The inset shows a WAXS image of an (SN)*_x_* crystal (COD 1008023) taken in the transmission mode. (g) Polarized optical micrograph of an (SN)*_x_* crystal grown out of the HOPG edge plane. (h) Raman spectra taken at different spots indicated in (g), red: (SN)*_x_* crystal on the HOPG surface; black: attached HOPG fragment on the (SN)*_x_* surface. (i) SEM image of fibers grown on HOPG platelets at the (hk0) edge surface. (j) SEM image of the (hk0) surface indicating HOPG sheets (upper part).

HOPG is frequently exfoliated, e.g., by mechanical treatment in order to produce mono- or multilayer graphene.^46^ Here, we want to keep the integrity of the HOPG specimen upon swelling for further direction-dependent magnetic measurements. There are several methods available for swelling of HOPG, such as electrochemical or solvent-based swelling.^24^ Standard solvents for HOPG swelling are NMP or its mixtures with DMF.^47^ For our purposes, it was found that swelling in NMP together with low-dose ultrasonication is the most suitable. NMP is a neutral, nonreactive solvent for the liquid-phase exfoliation of graphite. Furthermore, the content of separated graphite flakes after NMP treatment under our experimental conditions is small, and NMP can easily be removed in ultrahigh vacuum after the swelling procedure. The SEM image provided in Figure 3a was taken after this procedure. It shows a selected area of graphite sheets from an edge area after swelling in NMP, ultrasonication, and drying. Some typical kink-band striations can be observed.^48^ The intersheet areas of HOPG are now accessible to S_2_N_2_ vapor, followed by polymerization to (SN)_*x*_. This is demonstrated in Figure 3b in an early stage of the polymerization. Figures 3c shows a morphology where the HOPG sheets are further separated by the growth of (SN)_*x*_ nanowires. They grow in the crystallographic c-direction, i.e., along the direction of the strong diamagnetic field of HOPG. This seems to be one of the rare instances where a small magnetic field influences the polymer morphology during polymerization. There are some reports on the influence of strong magnetic fields on the morphology of diamagnetic polymeric materials.^49^ Most frequently, only effects of the magnetic field on polymerization kinetics^50^ and increases in molar mass during free-radical polymerization in magnetic fields are observed.^51^ The formation of (SN)_*x*_ nanowires can also be seen in an SEM image taken in the backscattering mode (see Figure 3d). Since nitrogen and sulfur have higher atomic numbers than carbon, the (SN)_*x*_ regions appear bright.

**Figure 3:**
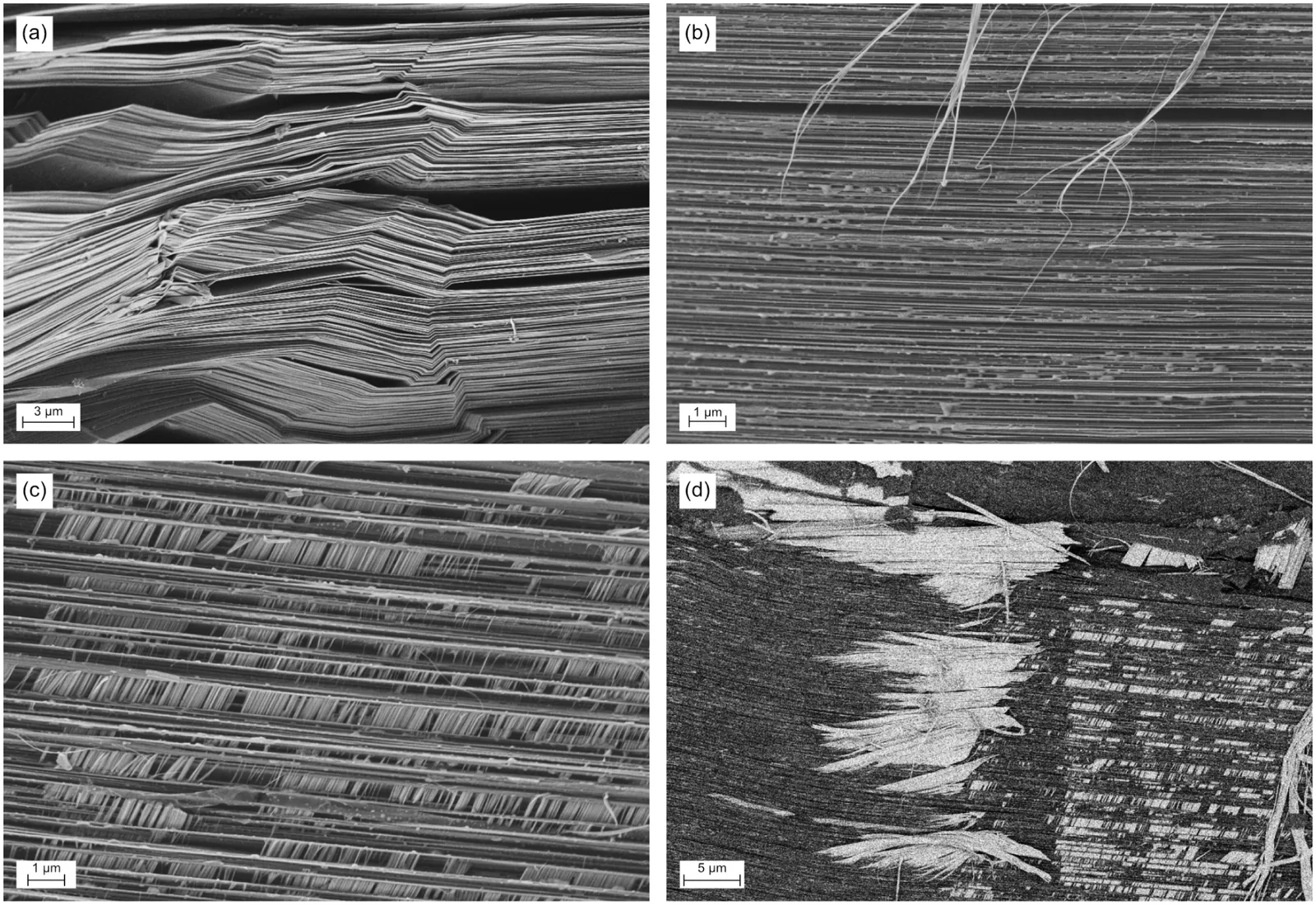
SEM images. (a) HOPG sheets after swelling in NMP and ultrasonication. (b) Early stage of (SN)*_x_* growth in the intersheet regions of HOPG after S_2_N_2_ treatment. (c) Regular nanowires grow in the intersheet regions at a later stage. (d) Image recorded in the backscattering mode.

In order to further verify (SN)_*x*_ formation within HOPG layers after NMP swelling, EDX measurements were carried out.

A typical SEM image is provided in Figure 4a. The elemental mapping obtained by EDX is provided in Figure 4b–e. The HOPG platelets contain mainly carbon, as expected. In contrast, the (SN)_*x*_ nanofibers are rich in nitrogen and sulfur. The complete sample surface exhibits some traces of oxygen. Altogether, the overlay image of the elemental mapping of Figure 4f reflects the SEM morphology image of Figure 4a. It should be noted that pristine HOPG also shows some traces of oxygen, but sulfur and nitrogen are completely absent (see Supporting Information Figure S5).

**Figure 4:**
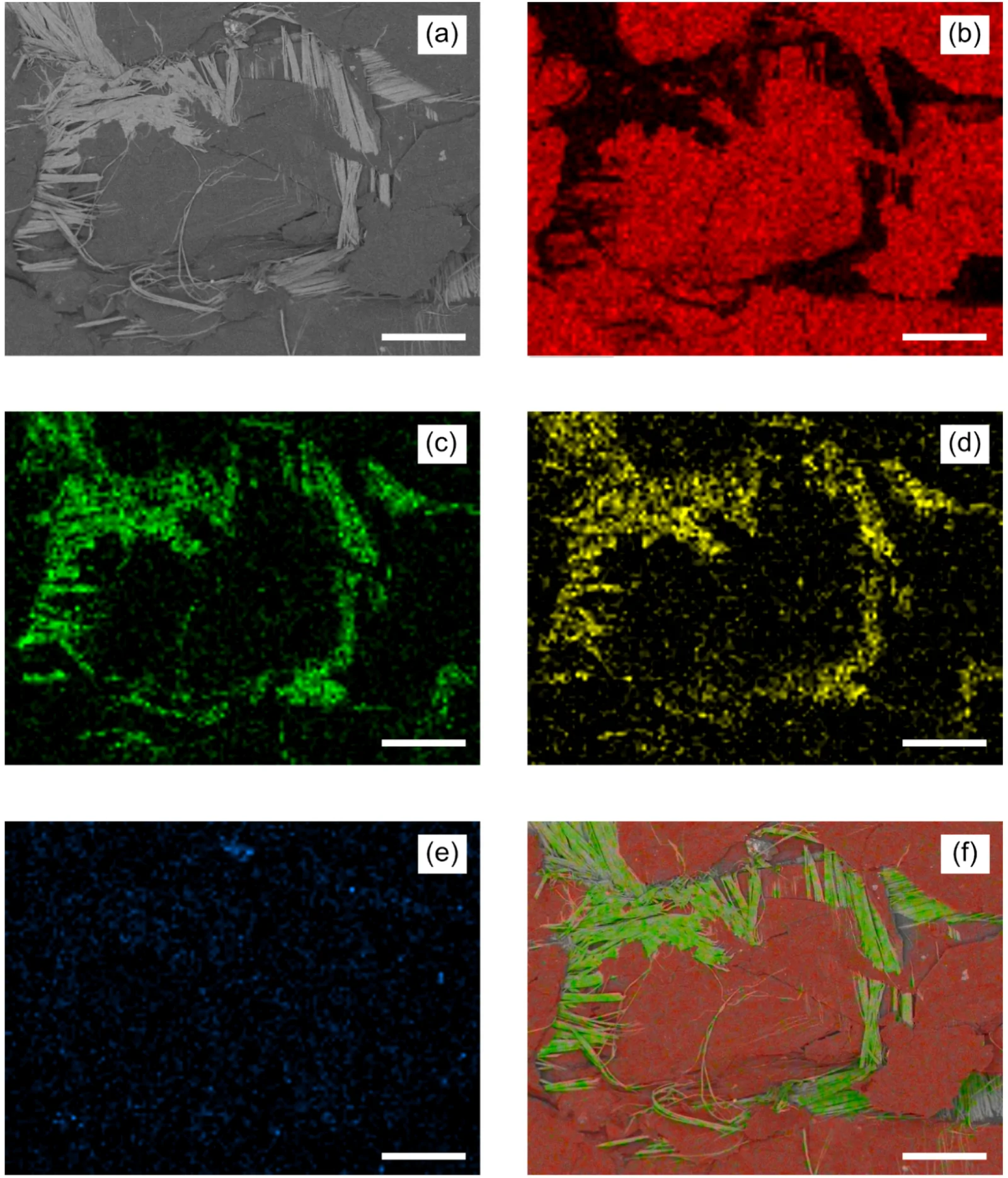
All scale bars are 10 μm. (a) SEM image of HOPG intercalated with (SN)_*x*_ after NMP swelling. (b–e) Elemental mapping obtained by EDX. (b) Carbon mapping. (c) Nitrogen mapping. (d) Sulfur mapping. (e) Oxygen mapping. (f) Overlay image.

Figure 5a,b clearly indicates that the ring-opening polymerization of S_2_N_2_ to (SN)_*x*_ is initiated at HOPG mosaic-crystal boundaries. This is different from the topochemical (SN)_*x*_ synthesis where the ring opening of S_2_N_2_ occurs within single crystals of the monomer.^37^ Several studies on carbon materials have shown that zigzag edges of 2D carbon materials are more reactive than armchair edges.^52^ The zigzag edges do not belong to a completely benzenoid graphene monolayer, and localized double bonds are present.^53^ Thus, zigzag edges are more reactive than armchair edges, where aromatic rings or localized double bonds can occur, resulting in lower reactivity in agreement with Clar’s rule.^54^ The initial (SN)_*x*_ nanowire growth has strong similarities with the Bitter pattern,^55^ which indicate magnetic domains in superconductors below their critical temperatures.^56,57^ Therefore, diamagnetism might be the reason for the occurrence of these structures, even in the case that the diamagnetism of the system under investigation is several orders of magnitude smaller than the susceptibility of superconductors below their critical temperature (see below). This might also be the reason that no crystals of (SN)_*x*_ are formed since the initial growth prevents the packing of parallel fibers into well-defined crystals. It should be mentioned that single crystals of (SN)_*x*_ in a crystallographic sense never occur due to the C2 rotation during the chain growth.^47^ This always leads to extensive twinning of different kinds already on the nm scale.^58,59^ The ring opening of S_2_N_2_ leads to the formation of a diradical,^60–62^ which probably attacks the isolated double bonds of the zigzag edges of HOPG.^63,64^ The edge regions of carbon materials are always more reactive compared to the center regions even when passivated with hydrogen or oxygen-containing groups.^65,66^ Thus, one would always expect (SN)_*x*_ growth parallel to the HOPG basal planes (see Figure 5c,d). The fact that (SN)_*x*_ nanowires grow perpendicular to the basal HOPG planes in the crystallographic c-direction might be caused by the influence of the diamagnetic magnetic field. This behavior has also been known by the alignment of liquid crystals in weak magnetic fields.^67^ The higher magnification of Figure 5e clearly shows that (SN)_*x*_ growth is initiated at HOPG terraces, indicated by yellow arrows. It can be seen that the growth of (SN)_*x*_ nanowires even deforms HOPG platelets, which is indicative of chemical bonds between HOPG and (SN)_*x*_ (see yellow arrows in Figure 5f). Thus, the (SN)_*x*_ nanowire growth occurs in the magnetic field direction detected in the secondary electron detector mode (Figure 5g). When the same image is taken in the backscattering mode, it is clear from the bright appearance of the nanowires that they are formed by (SN)_*x*_ (Figure 5h). This is due to the fact that sulfur and nitrogen are heavier elements compared to carbon.

**Figure 5:**
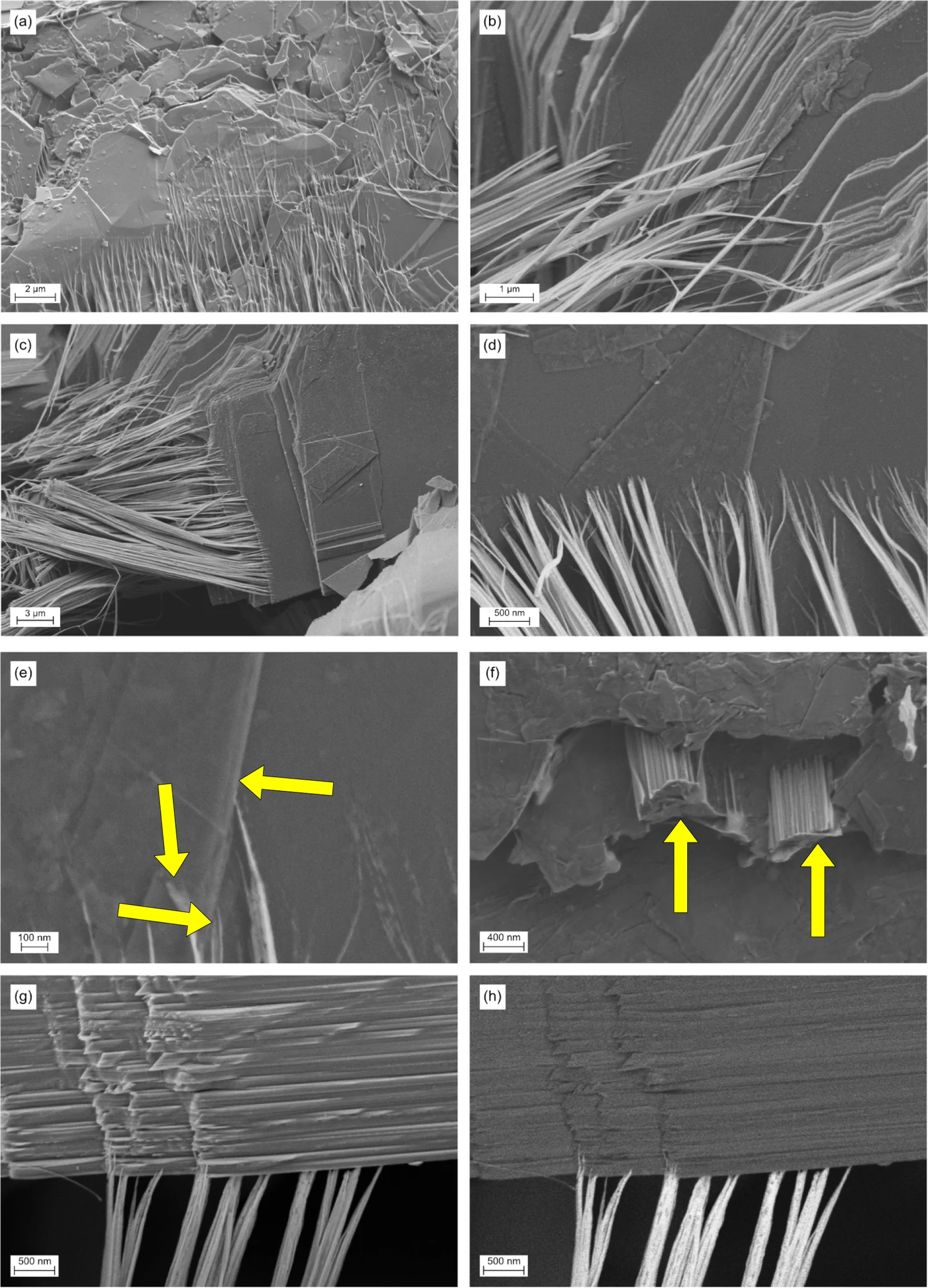
SEM images. (a) Growth of (SN)*_x_* nanowires on (hk0) faces of HOPG crystallites. (b) Higher magnification of nucleation sites of (SN)*_x_* nanowires. (c) Growth of (SN)*_x_* nanowires on the apparent (00l) surfaces. (d) Higher magnification image of the (SN)*_x_* nanowires on HOPG basal planes. (e) A higher magnification shows clearly that the (SN)*_x_* nanowire growth is initiated at HOPG terraces indicated by yellow arrows. (f) (SN)*_x_* nanowire growth deforms HOPG platelets (yellow arrows). (g) (SN)*_x_* nanowire growth in the magnetic field direction, detected in the secondary electron detector mode. (h) As in (g) but measured in the backscattering mode.

### Magnetic Properties

The π-orbitals of the carbon atoms are oriented along the c-direction of HOPG. The electrical conduction of HOPG is considered to be a quantum mechanical hopping process of electrons along the basal plane. There is only a slight overlap of the valence and conduction bands at the Fermi level (0.04 eV). Thus, HOPG is a typical semimetal with a charge-carrier concentration of ∼10^–4^ per C atom.^1^ Since an electrical current is always connected with a magnetic field, we focus on magnetic measurements as a function of the crystallographic a- or c-direction (also assigned as || and ⊥).^21^ The magnetic properties of HOPG result from circular currents above and beneath the single graphene layers. For the characterization of magnetic properties of materials, it is common to measure the magnetic susceptibility π, which is defined as^68^

1 M=χH

where *M* is the material’s magnetization and *H* is the applied external magnetic field. The susceptibility can be determined experimentally in a direct current (DC) field or in an alternating current (AC) field. Here, we discuss the AC susceptibility π_AC_, which is a complex quantity

2 $$\chi_{AC}=\chi^{\prime }-i\chi^{\prime \prime }$$

where the real part χ′ is related to the reversible magnetization process and the imaginary part χ″ is characterized by losses caused by irreversible processes and, in turn, energy absorption.^69^ The applied alternating magnetic field is given by

3 $$H\left(t\right)=H_{0}+h\cos\left(2\pi ft\right)$$

where *H*_0_ is a static bias field, *h* is the amplitude of the applied magnetic AC-field, and *f* is the frequency. The magnetization *M­(t)* is then given by

4 $$M\left(t\right)=M_{0}+m\cos\left(2\pi ft-\theta \right)$$

with *M*_0_ being the bias magnetization, *m* being the amplitude of the AC magnetization, and θ being the phase shift. Thus, the real and the imaginary part of the susceptibility can be written as

5 $$\chi^{\prime }=m\cos\theta /h$$

6 $$\chi^{\prime \prime }=m\sin\theta /h$$

The intercalation of HOPG with (SN)_*x*_ leads to an isotropication of the magnetic properties of the system under consideration. This can be seen in detail in Figure 6 showing AC susceptibility measurements. Figure 6a shows that $\chi_{∥}^{\prime }$ is negative and increases with temperature. There is no difference for $\chi_{∥}^{\prime }$ of HOPG and HOPG + (SN)_*x*_. The situation is different when $\chi_{⊥}^{\prime }$ is measured (Figure 6b). The real part of impedance $\chi_{⊥}^{\prime }$ for neat HOPG is very small and negative, as also observed for neat (SN)_*x*_.^70^ In contrast, significant diamagnetism is observed for HOPG + (SN)_*x*_. This is a clear indication that an electrical current flows along the (SN)_*x*_ nanowires perpendicular to the HOPG sheets. It results in a magnetic field perpendicular to the flowing current. The same behavior has been observed when Yb or Ca ions are intercalated into HOPG, and the superconducting transition temperature is observed.^71^ If (SN)_*x*_ simply increased the graphene layer distance, an opposite effect would be expected. Similar results are obtained for the imaginary part of the susceptibility. $\chi_{∥}^{\prime \prime }$ is very similar for neat HOPG and HOPG + (SN)_*x*_ as can be seen in Figure 6c. In contrast, $\chi_{⊥}^{\prime \prime }$ is close to zero for neat HOPG and neat (SN)_*x*_ (Figure 6d). Only for the HOPG + (SN)_*x*_ system can measurable positive values be observed. It decreases up to about 50 K and then remains nearly constant.^19^

**Figure 6:**
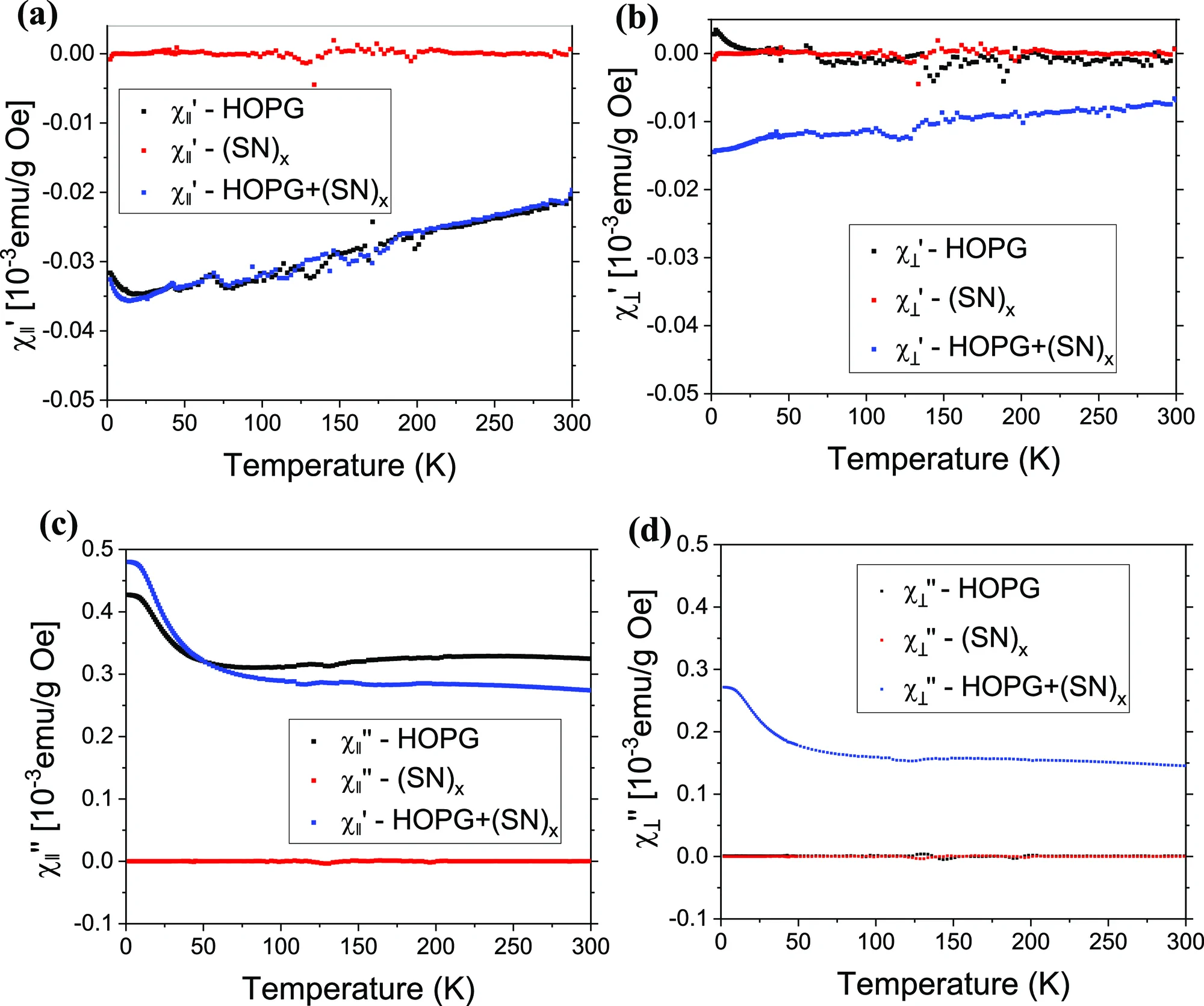
Direction-dependent AC measurements of neat (SN)*_x_*, neat HOPG, and HOPG intercalated with (SN)*_x_*. (a) The real part $\chi_{∥}^{\prime }$ of the susceptibility in the crystallographic c-direction and (b) $\chi_{⊥}^{\prime }$ in the crystallographic basal plane. (c) The imaginary part $\chi_{∥}^{\prime \prime }$ parallel to the c-direction and (d) $\chi_{⊥}^{\prime \prime }$ in the basal plane.

## Conclusion

It has been demonstrated that (SN)_*x*_ can successfully intercalate into HOPG galleries by *in situ* polymerization of S_2_N_2_ in the gas phase. For this purpose, HOPG needs a pretreatment such as solvent swelling together with ultrasonic treatment. The polymerization of S_2_N_2_ to (SN)_*x*_ is initiated at HOPG defect structures. In contrast to the bulk polymerization of S_2_N_2_, (SN)_*x*_ forms mostly single nanofibers and does not form large fibrous crystals. The polymerization takes place mainly in the edge region of HOPG. When polymerized on the 00l surfaces, larger (SN)_*x*_ crystals are similar to bulk crystals. The crystals are not mechanically stable when attached to HOPG surfaces and can easily be removed. The incorporation of (SN)_*x*_ nanowires at the HOPG edges has a stunning influence on the diamagnetism of HOPG. Strong diamagnetism of HOPG + (SN)_*x*_ is observed parallel to the graphene layers, in contrast to native HOPG, where this value is very small. This is schematically shown in Figure 7.

**Figure 7:**
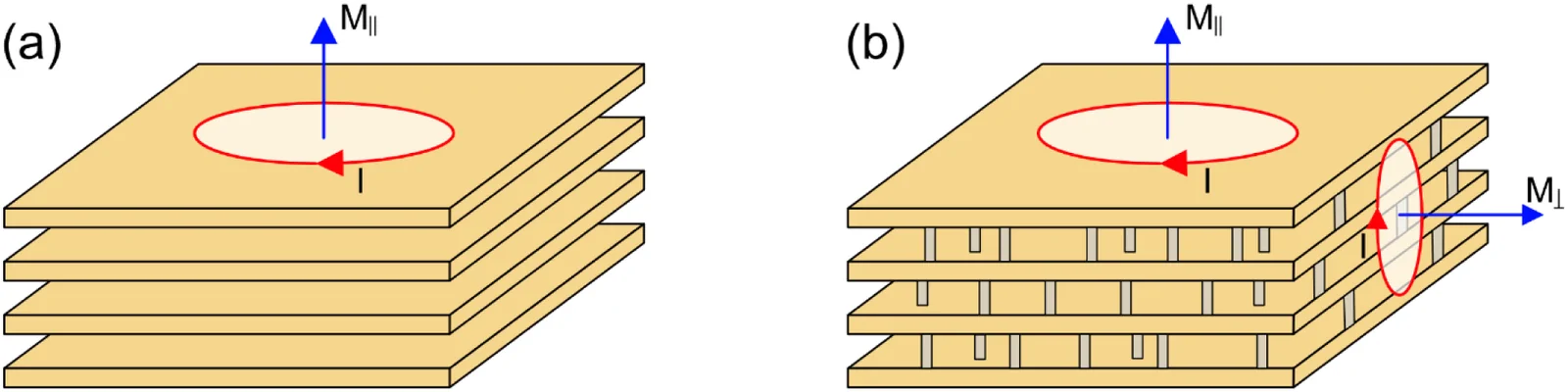
(a) Diamagnetic moment in pure HOPG and (b) diamagnetic moments in HOPG + (SN)_*x*_ with (SN)_*x*_ short circuits between HOPG sheets.

The circular basal plane current in HOPG, shown in Figure 7a, leads to a strong diamagnetic moment in the c-direction, whereas the diamagnetic moment in the direction of the basal plane is very small. The situation is different in the case of HOPG + (SN)_*x*_. The HOPG mosaic crystallites still have a thickness of about 30 nm. With a distance between two graphene layers of about 3.35 Å, we have approximately 90 single graphene layers within one sheet. Thus, the diamagnetic moment in the c-direction is not significantly influenced by (SN)_*x*_ intercalation. However, the diamagnetic moment in the basal plane is strongly increased. These results may contribute to applications of HOPG in the field of quantum wires,^72,73^ magnetic levitation,^74^ and electron bridge engineering.^75–77^

## Experimental Section

### Materials and Synthesis

HOPG grade 2 (mosaicity 0.8 ± 0.2°) was purchased from Plano GmbH (Wetzlar, Germany). The specimen of 10 mm × 10 mm × 1 mm was cut into four pieces using a scalpel. Swelling was carried out in *N*-methyl pyrrolidone (NMP; Carl Roth GmbH, Karlsruhe, Germany) at 60 °C for 12 days. At the beginning and at the end, the samples were ultrasonicated for 3 min at 45 kHz (Elma Schmidbauer GmbH, Singen, Germany). Under these experimental conditions, the integrity of the HOPG specimen was preserved. ^15^N-labeled S_4_N_4_ was synthesized in our laboratory and had an isotope ratio of ^14^N:^15^N of 57:43 mol %.^36^ The solid-state polymerization of (SN)_*x*_ followed the procedure reported by Labes et al.,^26^ where in a first step S_4_N_4_ is converted over silver wool (Carl Roth GmbH, Karlsruhe, Germany) as a catalyst to S_2_N_2_. Four pieces of swollen HOPG were treated for 2 days under vacuum at 4.4 × 10^–3^ mbar at room temperature in order to remove residual NMP. Then, the four pieces were brought into the S_2_N_2_ gas stream for 7 h at 0 °C and then kept under vacuum for another 5 days at room temperature (Figure 8; for full details, see Supporting Information, Figure S3). Finally, the samples were kept for 5 weeks in vacuum at room temperature. This results in the formation of (SN)_*x*_ crystals at the glass wall, together with some (SN)_*x*_ crystals on the HOPG surface, and (SN)_*x*_ intercalated in HOPG.

**Figure 8:**
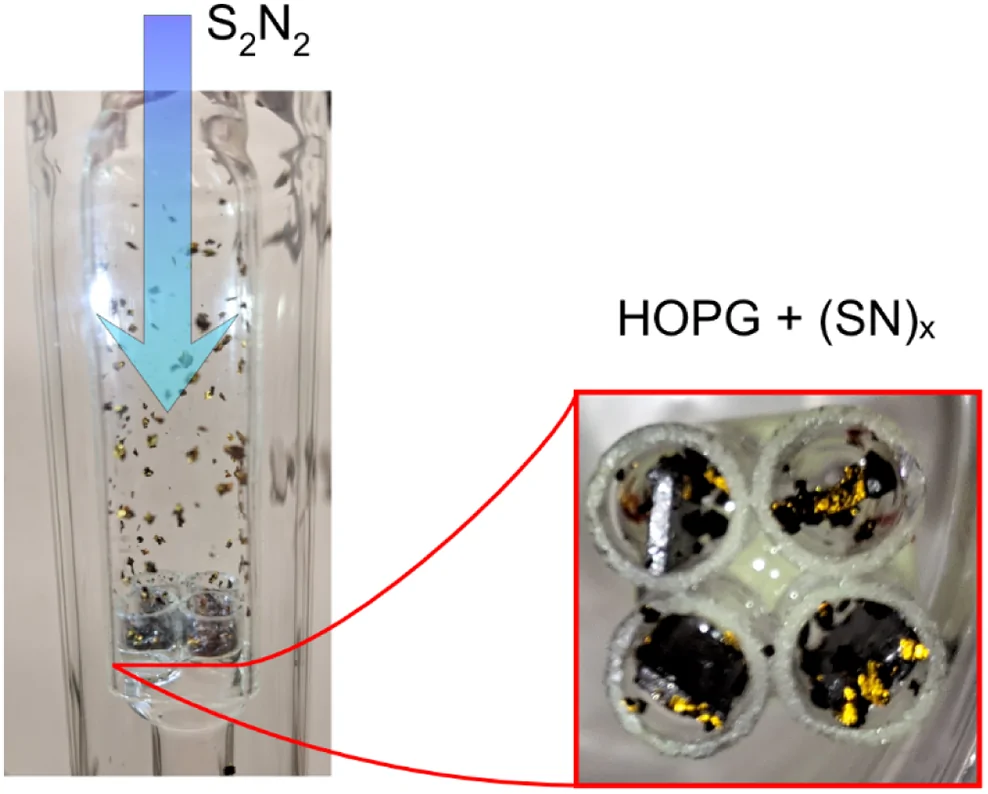
Part of the polymerization apparatus where (SN)*_x_* is synthesized. Neat (SN)*_x_* crystals are found on the inner glass wall and on the HOPG specimens. Additionally, (SN)*_x_* is formed between HOPG sheets (see below). The complete apparatus is shown in Supporting Information, Figure S3.

### Characterization

Scanning electron microscopy (SEM) was carried out with a Gemini SEM 500 (Carl Zeiss Microscopy GmbH, Oberkochen). An acceleration voltage of 2 kV was applied. The detector employed either secondary electrons or backscattered electrons. For energy-dispersive X-ray analysis (EDX), an X-MAX EXTREM detector from Oxford Instruments (High Wycombe, UK) was used. An acceleration voltage for local EDX analysis of 3 kV was used.

Confocal Raman microscopy was performed with a Renishaw inVia Raman microscope (Renishaw plc, UK). A Leica DMI 3000 inverted microscope (Leica Microsystems GmbH, Germany) was equipped with a charge-coupled device (CCD) camera. The Cobolt CW DPSS laser had a wavelength of 532 nm and a grating of 1800 lines mm^–1^. Before starting the experiments, the Raman spectrometer was calibrated to a silicon reference peak, which was adjusted to 520.4 cm^–1^. Every spectrum was recorded between 150 and 3000 cm ^–1^ with 1.0% laser intensity and 10 s of integration time for all samples.

Magnetometer: Magnetic measurements were performed with the ACMS option of a Quantum Design PPMS-9. Prior to measurements, all homopolymer (SN)_*x*_ crystals were removed from the sample surfaces. Between ∼30 and ∼70 mg of each sample was placed in the sample holder, which had only a small diamagnetic contribution to the measured susceptibilities. For each sample, a background measurement was subtracted. The temperature-dependent moment was measured at an external field in the temperature range of 2–300 K, applying field-cooled (FC) conditions. The frequency for AC measurements was 1000 Hz. The amount of (SN)_*x*_ in the HOPG + (SN)_*x*_ sample was below 1 wt % as obtained by thermogravimetry (see Supporting Information, Figure S4)

2D-WAXS measurements were performed at room temperature with an Incoatec IμS (Geesthacht, Germany) equipped with a microfocus source and a monochromator for Cu–K_α_ radiation (λ = 1.5406 Å). 2D scattering patterns were recorded with a Vantec 500 2D detector (Bruker AXS, Karlsruhe). 1D-WAXS experiments were performed with an Empyrean Diffraction System from PANalytical.

## Supplementary Material



## Notes

Polarized optical microscopy and X-ray transmission measurements of HOPG + (SN)_*x*_; Raman spectroscopy of neat HOPG; synthesis setup; thermogravimetry of HOPG + (SN)_*x*_; SEM images and EDX spectrum of pristine HOPG (PDF)

## Abbreviations

AC: alternating current
DC: direct current
DMF: dimethylformamide
fwhm: full width at half-maximum
HOPG: highly oriented pyrolytic graphite
NMP: *N*-methyl pyrrolidone
RT: room temperature
SEM: scanning electron microscopy
S_2_N_2_: disulfur dinitride
S_4_N_4_: tetrasulfur tetranitride
(SN)_*x*_: poly­(sulfur nitride)
WAXS: wide-angle X-ray scattering
