# Supplementary material for: Poly(sulfur nitride) Nanowires as Short Circuits between Highly Oriented Pyrolytic Graphite Sheets
Source: Langmuir. 2026 Sep 2;42(36):26384–95. doi: 10.1021/acs.langmuir.6c03183 (PMC13585138; doi:10.1021/acs.langmuir.6c03183)

Supporting Information (SI)

Poly(sulfur nitride) Nanowires as Short Circuits between Highly Oriented Pyrolytic Graphite Sheets

Jörg Kressler[a,], Karsten Busse[a], Julian Schoch-Radicke[a], Oleksandr Dolynchuk[a], Stefan G. Ebbinghaus[a], Frank Syrowatka[b]*

a) Faculty of Natural Science II, Martin Luther University Halle-Wittenberg, D-06120 Halle (Saale), Germany

b) Interdisciplinary Center of Materials Science, Martin Luther University Halle-Wittenberg, 06099 Halle (Saale), Germany

*Corresponding author email address: joerg.kressler@chemie.uni-halle.de

Table of content:

S2 Polarized optical microscopy images of HOPG + $(SN)_x$ (Figure S1)

S3 Raman spectrum of neat HOPG (Figure S2)

S4 Experimental setup for synthesis (Figure S3)

S5 Thermogravimetry of HOPG + $(SN)_x$ (Figure S4)

S6 SEM images of pristine HOPG and EDX spectrum (Figure S5)

Figure S1

Polarized optical microscopy image of a 5 mm x 5 mm x 1mm sample of HOPG + $(SN)_x$. The red dots of the left image indicate the places where X-ray transmission measurements below are taken. The middle image is the front side and the right image shows the backside

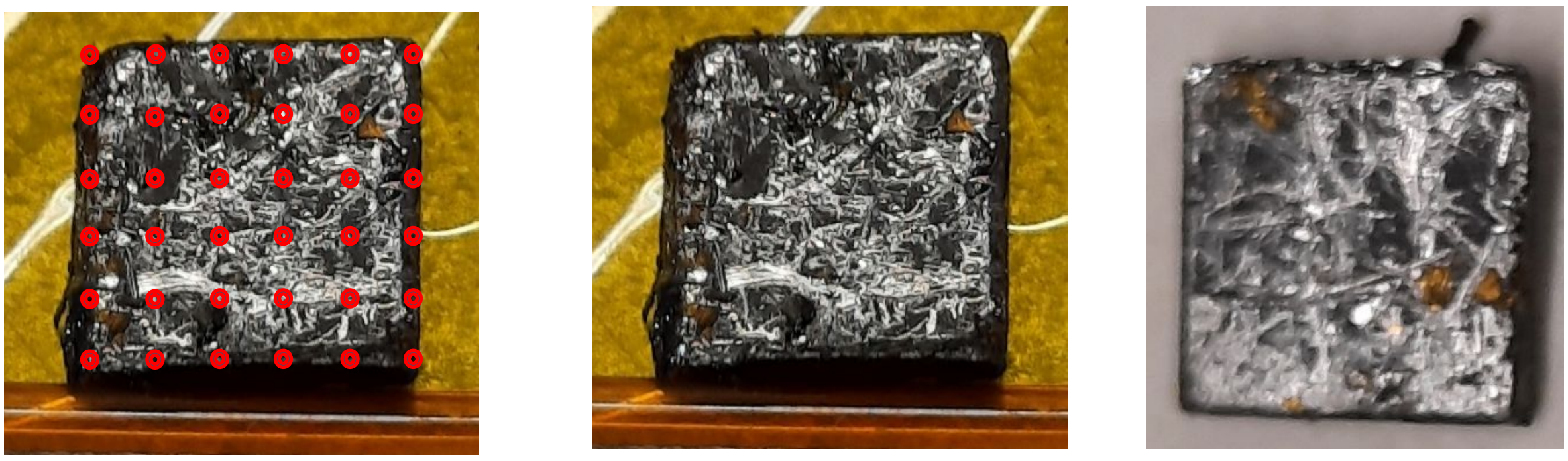

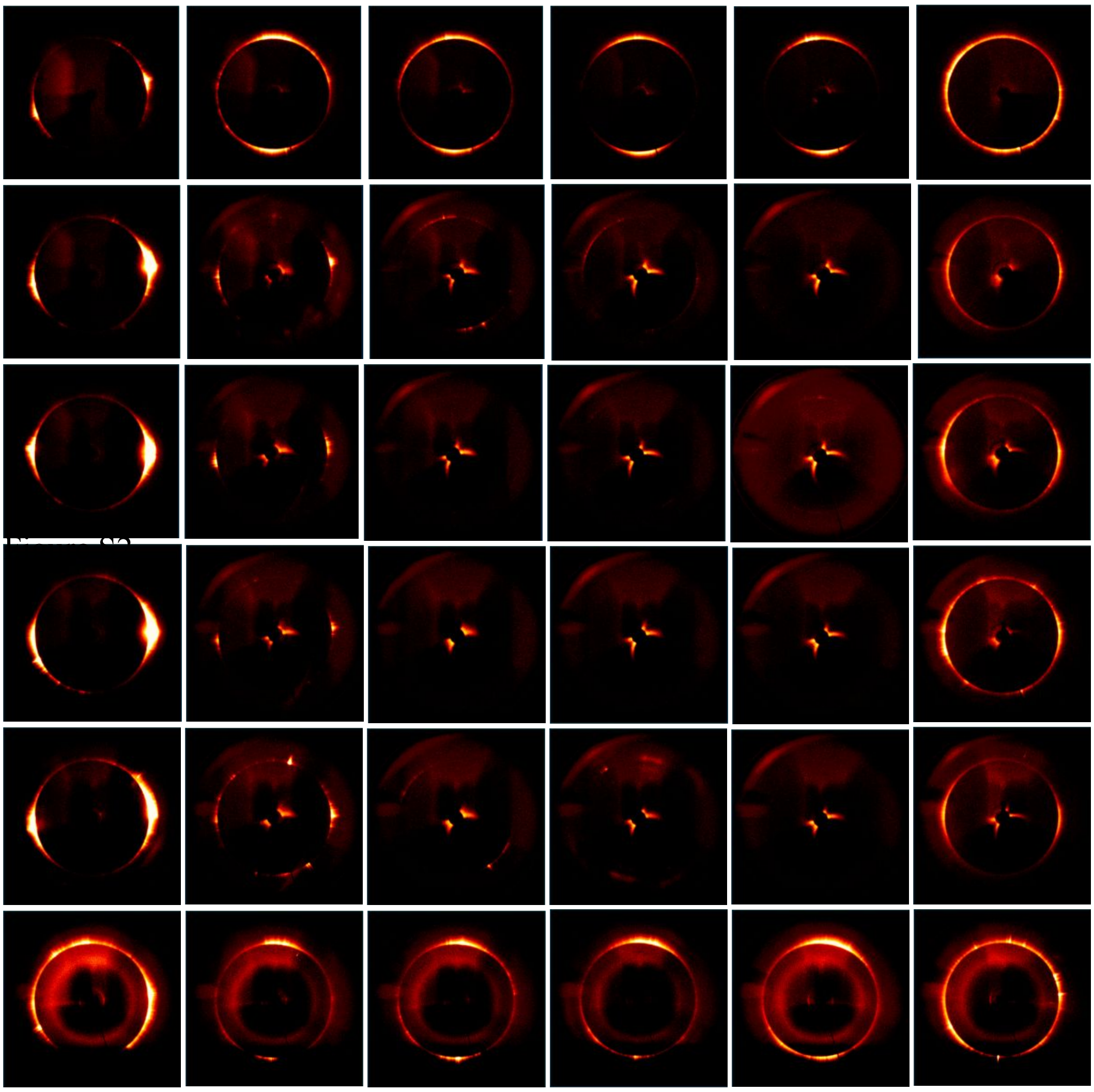

Figure S2

Raman spectrum of neat HOPG

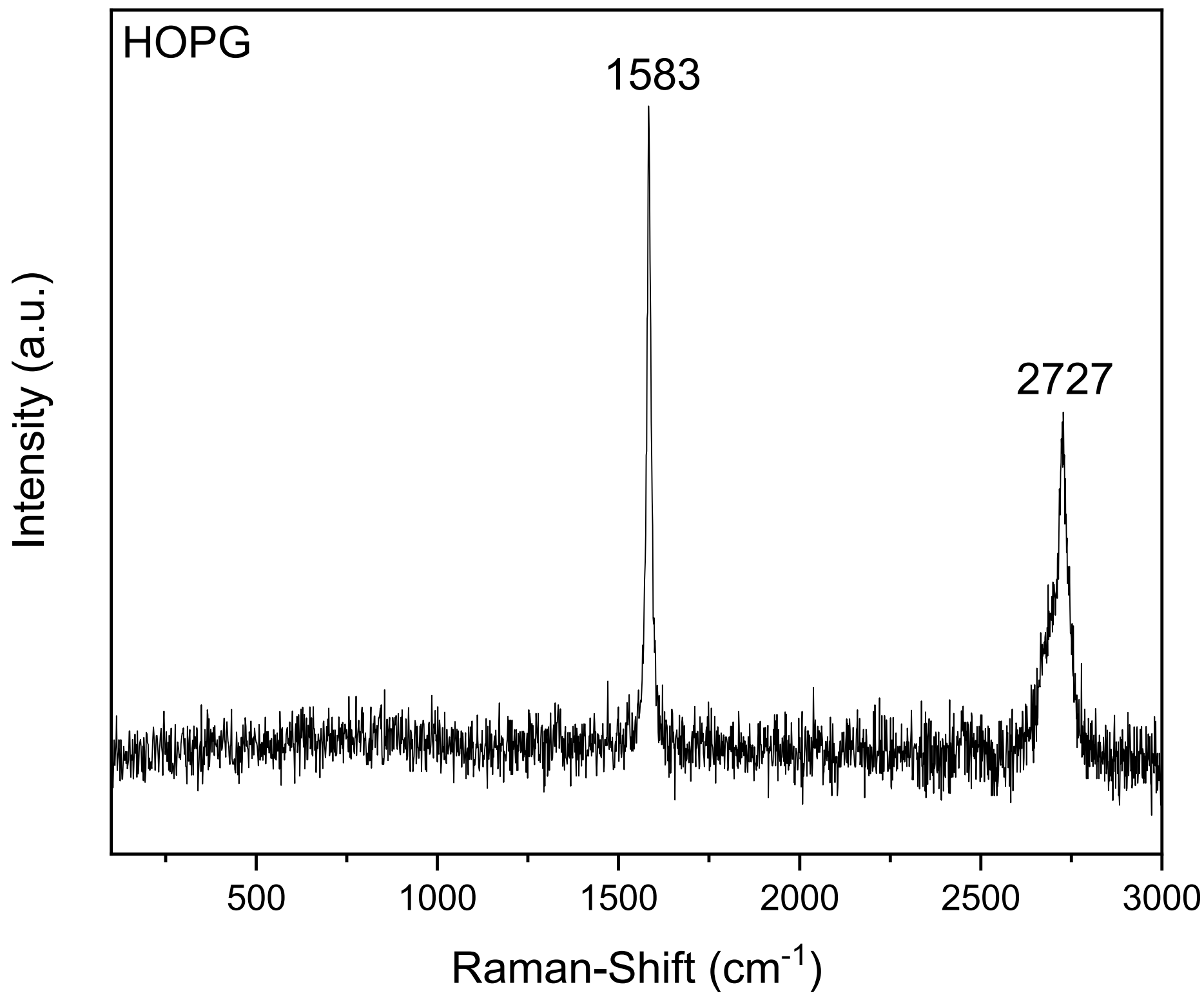

Figure S3

Experimental setup

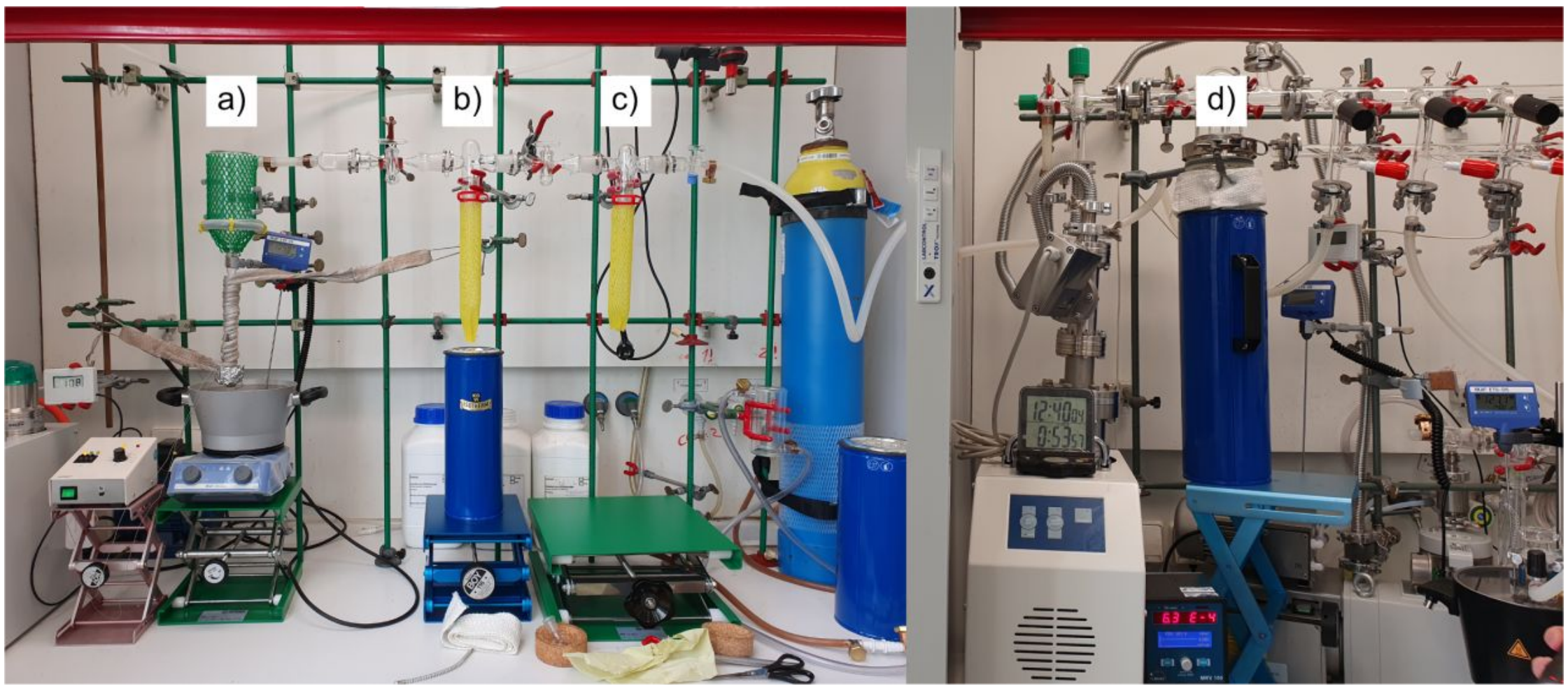

a) – less than 1 g S_4N_4 in an oil bath at 95°C, the column is heated to 265°C, on top is a vessel filled with chloroform and cooled with dry ice

b) – here the polymerization took place, cooled by water/ice

c) – cooling by liquid nitrogen

d) – Schlenk line with turbo pump equipment, and the vacuum was in the range of 10^{-3} mbar

Figure S4

Thermogravimetry of HOPG + $(SN)_x$ in air measured with a heating rate of 10 K/min. The weight loss at about 160°C indicates the $(SN)_x$ decomposition. The following weight increase is related to the formation of graphene oxide.

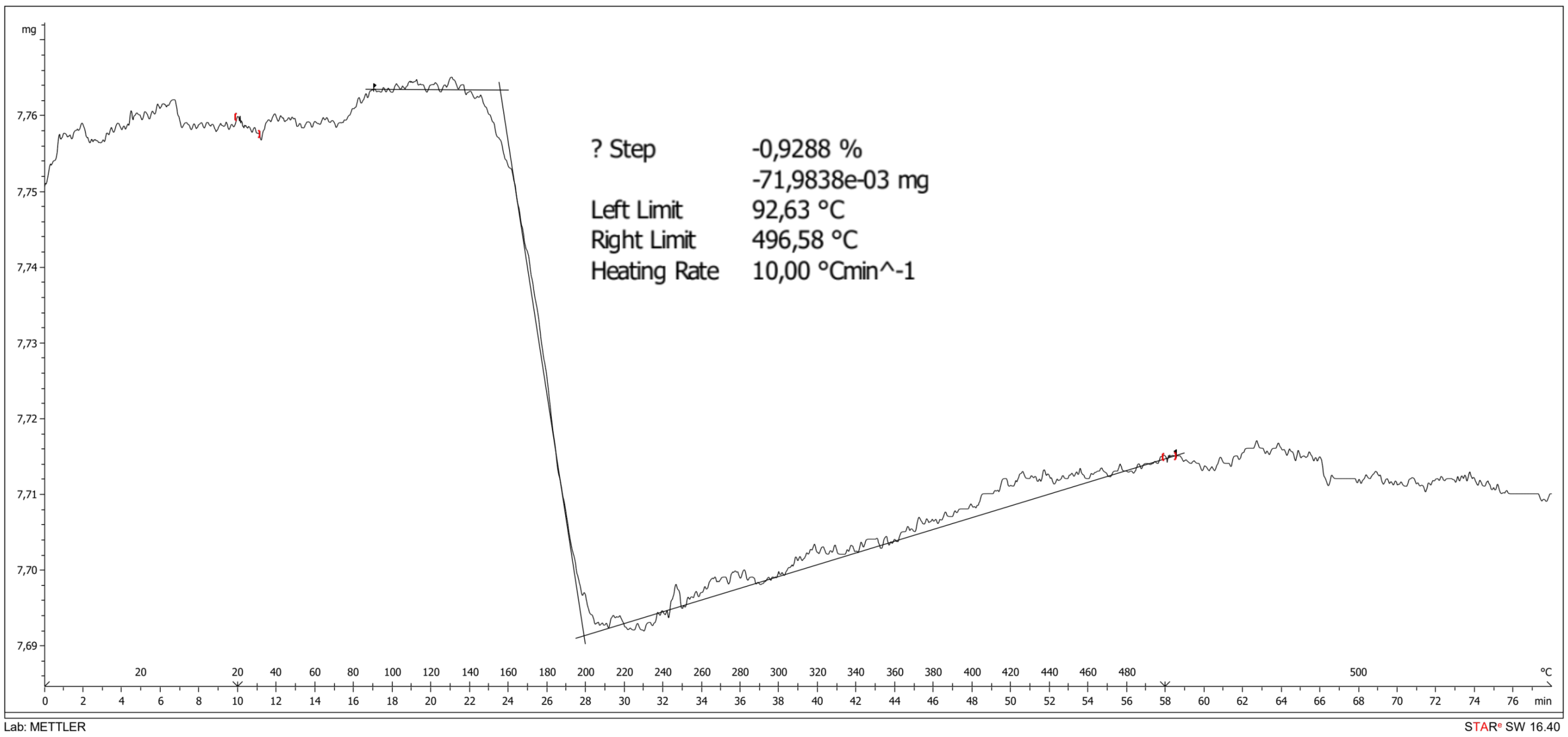

Figure S5

SEM images of pristine HOPG (S5a, c-f) and EDX spectrum (S5b) at the position indicated in Fig. S5a.

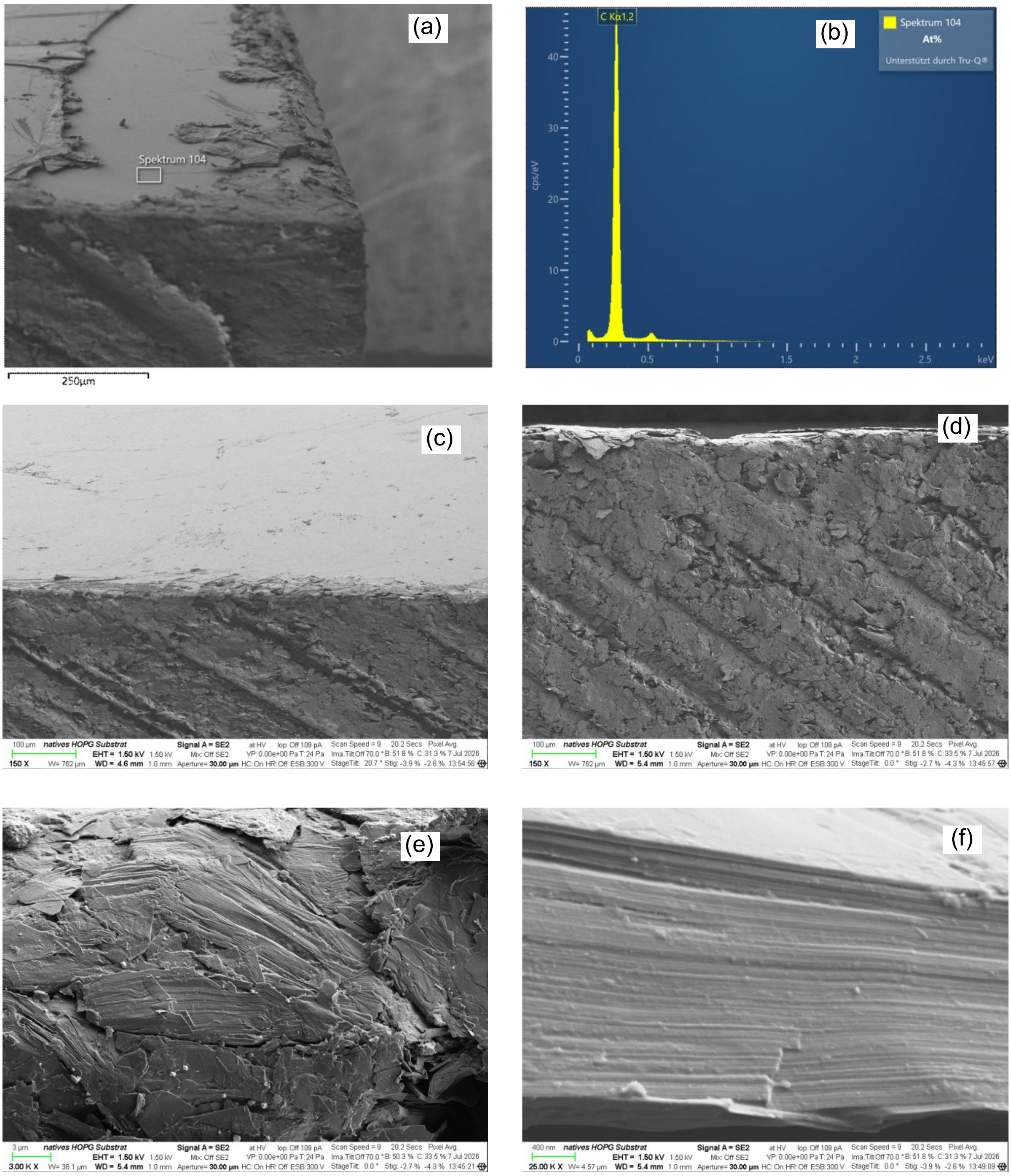

Supplement: Supplementary file 1 [file la6c03183_si_001.pdf]
